# Food Access and Diet Quality Are Associated with Quality of Life Outcomes among HIV-Infected Individuals in Uganda

**DOI:** 10.1371/journal.pone.0062353

**Published:** 2013-04-18

**Authors:** Tia Palermo, Rahul Rawat, Sheri D. Weiser, Suneetha Kadiyala

**Affiliations:** 1 Stony Brook University (SUNY), Program in Public Health/Department of Preventive Medicine, Stony Brook, New York, United States of America; 2 Poverty, Health, and Nutrition Division, International Food Policy Research Institute (IFPRI), Washington, D.C., United States of America; 3 Division of HIV/AIDS, San Francisco General Hospital, UCSF, San Francisco, California, United States of America; UNAIDS, Switzerland

## Abstract

**Background:**

Food insecurity is associated with poor nutritional and clinical outcomes among people living with HIV/AIDS. Few studies investigate the link between food insecurity, dietary diversity and health-related quality of life among people living with HIV/AIDS.

**Objective:**

We investigated whether household food access and individual dietary diversity are associated with health-related quality of life among people living with HIV/AIDS in Uganda.

**Methods:**

We surveyed 902 people living with HIV/AIDS and their households from two clinics in Northern Uganda. Health-related quality of life outcomes were assessed using the Medical Outcomes Study (MOS)-HIV Survey. We performed multivariate regressions to investigate the relationship between health-related quality of life, household food insecurity and individual dietary diversity.

**Results:**

People living with HIV/AIDS from severe food insecurity households have mean mental health status scores that are 1.7 points lower (*p*<.001) and physical health status scores that are 1.5 points lower (*p*<.01). Individuals with high dietary diversity have mean mental health status scores that were 3.6 points higher (*p*<.001) and physical health status scores that were 2.8 points higher (*p*<.05).

**Conclusions:**

Food access and diet quality are associated with health-related quality of life and may be considered as part of comprehensive interventions designed to mitigate psychosocial consequences of HIV.

## Introduction

Among people living with HIV (PLHIV), health related quality of life (HRQoL) is particularly important in the management of the disease, due to its chronic nature and the disease's myriad mental, emotional and physical manifestations over its course. HRQoL refers to how well a person functions and to his or her perceptions of well-being in the physical, mental, and social domains of life–all distinct areas that are influenced by a person's experiences, beliefs, and expectations [1], [2]. There is substantial and consistent evidence that HRQoL is negatively associated with indicators of HIV disease progression, including CD4 T- lymphocytes and increases in viral load, among both antiretroviral therapy (ART)-naïve and those on ART [3]–[6].

Food insecurity is highly prevalent in Uganda and other countries in sub-Saharan Africa, especially among PLHIV [7], . Defined as “the limited or uncertain availability of nutritionally adequate, safe foods or the inability to acquire personally acceptable foods in socially acceptable ways,” [9], food insecurity is linked to adverse health outcomes, including cardiovascular risk factors and diabetes [10], poor self-reported health [11], [12], and nutrient inadequacy [13]. Furthermore, it is associated with lower HRQoL in general populations and among those with chronic disease or disability [12], [14], [15]. Food insecurity is now recognized as fundamental to the four core pillars of a holistic response against HIV–prevention, care, treatment and mitigation [16]–[18]. It is strongly associated with increased behavioral risk of HIV transmission [16], [19]–[22], reduced access to HIV treatment and care [16], decreased adherence to antiretroviral therapy (ART) [21], [23], poor nutritional status [24], and worse clinical outcomes among HIV-infected individuals [22], [25], [26]; and relatedly, clinical fasting is associated with adverse antiretroviral pharmacokinetics [27], [28].

Like HRQoL, food security is a multidimensional construct. The four dimensions embodied by the definition of food security within the United Nations framework include (1) physical availability of food, (2) economic and physical access to food, (3) food utilization, and (4) stability of the other three dimensions over time [29]. Because of the many facets of the construct, there is lack of clarity in the literature on the dimensions of food security critical for the HRQoL of PLHIV. In this study we focused on aspects of the second and third dimensions, specifically food access and diet diversity. Studies have reported a strong association between HRQoL and several socioeconomic characteristics [5], [30] and health related indicators [4], [6], [30] in resource poor settings. But the investigation of the relationship between food security and HRQoL among PLHIV is just beginning to emerge [6], [16], [31]. Available evidence, albeit scant, suggests food insecurity and poor nutritional status to be important risk factors for poor mental and physical health. Studies to date examined only how the access and experiential dimensions of food security are associated with measures of HRQoL [6], [31]. None to date have examined the relationship between one of the most central dimensions of food security–diet quality–and its association with HRQoL among populations with chronic diseases, including PLHIV. Diverse measures of diet quality have been associated with nutrient adequacy [32], [33], and several health and nutrition outcomes [24], [34], including all-cause mortality [35], in developed and developing countries.

Given the limited existing research investigating the relationship between various dimensions of food security and HRQoL among PLHIV, we assessed whether food access and diet quality are associated with HRQoL among PLHIV in Uganda. We hypothesized that HRQoL outcomes would be negatively correlated with household food insecurity and positively correlated with diet quality.

## Materials and Methods

### Ethics statement

The study protocol was approved by the ethics boards of The AIDS Support Organization (TASO), the Uganda National Council on Science and Technology (UNCST), and the International Food Policy Research Institute (IFPRI). Interviewers read consent forms to participants and participants signed to give informed consent.

### Data and Sample

Data used in this study came from a baseline survey of 902 PLHIV and their households recruited into a study evaluating the impact of a World Food Programme (WFP) supported food assistance program in Uganda. The study was conducted in two districts in Uganda (Soroti and Gulu) among PLHIV registered with TASO between August 2008 and September 2009. TASO has supported over 200,000 individuals directly since its inception in 1987, and is the largest indigenous non-governmental organization in Uganda with 11 service centers providing HIV prevention, care, and treatment services [24]. At the time recruitment began for the study, the TASO center in Soroti served approximately 8,000 HIV-infected clients and the center in Gulu served approximately 12,000 clients. Both Soroti and Gulu districts are in Northern Uganda, have experienced recent conflict, and have large populations of displaced persons. The study was nested within the prevailing WFP and TASO operational context. The Gulu district where WFP was operational when the study was initiated served as the intervention district and Soroti, where WFP was not operational, served as the comparison district. Four hundred fifty one adult HIV-positive individuals were recruited from each district based on the following eligibility criteria: 1) eligibility for receipt of a WFP monthly household food basket, based on WFP's poverty assessment criteria, 2) non receipt of food assistance in the previous 12 months, 3) CD4 count between 200 and 459 cell/mm^3^, and 4) ART naïve. The WFP poverty assessment tool was administered by TASO personnel and included the following domains: household composition, employment status, income, housing characteristics, basic food sources and consumption, household expenditures, and access to services. The WFP monthly household food basket comprises corn, soy blended flour, vegetable oil, pulses, and maize meal or rice.

### Measures

The main outcome in this analysis, HRQoL, was operationalized by the physical health summary (PHS) and the mental health summary (MHS) scores of the Medical Outcomes Study (MOS)-HIV Health Survey [1], [36]. The MOS-HIV Survey and its variant short forms (SF-12, SF-20, SF-32) have been implemented widely in sub-Saharan Africa [3], [31], [37], [38] and elsewhere [36],[39]–[44], have been translated into various languages, and have undergone cultural adaptation [45]. This instrument has been shown to have good reliability and validity [45], [46], including among HIV-infected individuals in Uganda [47], where the instrument was first culturally adapted for use in rural Africa [3].

The MOS-HIV survey consists of questions which assess 11 dimensions of health-related quality of life, including general health perceptions, physical functioning, role functioning, pain, social functioning, mental health, energy, health distress, cognitive functioning, quality of life, and health transition (Table 1); ten of these were used to create summary scores described in more detail below. Using the 37 questions included in our survey, we scored subscales for each of ten dimensions and created the two summary scores as outlined in the MOS-HIV User's Manual [1]. Briefly, the subscales were scored as summated rating scales ranging from 0 to 100, where a higher score indicates better health or functioning.

**Table 1:** Summary of questions used to construct MOS subscales.

Subscale	Survey items	Range of raw score	Transformed mean score	SD
Physical functioning	Does current health limit in the following activities:	6–18	82.33	17.76
	Vigorous activities			
	Moderate activities			
	Walking uphill or climbing stairs			
	Bending, lifting objects, or kneeling			
	Walking a distance			
	Eating, dressing, bathing			
Role functioning	Health prevents from working at a job, doing work around the house or attending school	2–4	56.67	21.63
	Limited in the kinds or amounts of work, housework or school work because of health			
Social functioning	In past 30 days, health limits social activities, like visiting with friends or family	1–6	82.67	22.49
Cognitive functioning	In past 30 days:	4–24	73.27	19.11
	Had difficulty reasoning and making decisions			
	Forgot things that happened recently			
	Had trouble keeping attention on any activity for long			
	Had difficulty doing activities involving concentration and thinking			
Pain	How much bodily pain in past 30 days	2–11	58.50	19.58
	How much did pain interfere with normal work in past 30 days			
Mental health	In past 30 days, felt:	5–30	65.21	16.32
	Nervous			
	Calm and peaceful			
	Depressed			
	Happy			
	Couldn't be cheered up			
General health	In general, health is…(excellent/very good/good/fair/poor)	5–25	32.29	24.93
	True/false:			
	Somewhat ill			
	Healthy as other people			
	Health is excellent			
	Feeling bad recently			
Vitality	In past 30 days, felt:	4–24	60.60	16.79
	Full of life/energy			
	Without energy			
	Tired			
	Had enough energy to do things			
Health distress	In past 30 days, felt:	4–24	82.52	15.78
	Weighed down by health problems			
	Discouraged by health problems			
	Despair over health problems			
	Afraid because of health			
Quality of life	Quality of life during past 30 days/how things have been going	1–5	47.22	18.37
Health transition	Compared to 30 days ago, how would rate:	1–5	44.35	17.60
	Physical health now			
	Emotional condition now			
	Ability to care for and provide for children and family members now			

Physical health summary scores (PHS) and mental health summary scores (MHS) were developed using factors derived from factor analyses conducted by Revicki et al. [44]. For the PHS score, the subscales for physical function, pain, and role functioning contributed most strongly; and for the MHS score, subscales that contributed most strongly include mental health, health distress, quality of life, and cognitive function. Finally, the summary measures (MHS and PHS) were transformed to have a mean of 50 and a standard deviation of 10. Subscales representing the 11 dimensions (including health transition, which as per the manual, was not included in creating PHS and MHS measures) werealso used as individual outcome variables in our analysis. We assessed internal consistency of the different subscales using Cronbach's alpha.

The main independent variables of interest were household food insecurity and individual dietary diversity. Household food insecurity is measured using the Household Food Insecurity Access Scale (HFIAS), which has been validated in several countries [48]. The HFIAS was created from nine questions on experiential food insecurity (access), including experiencing anxiety and uncertainty about food supply, altering diet quality, and reducing quantity of food consumed. For each of the nine questions, response options included: 0 = no, 1 = rarely, 2 = sometimes, 3 = often. Scores on the HFIAS ranged from 0 to 27 and higher scores indicate more food insecurity. For analysis purposes, we dichotomized this variable [ = 1 if severely food insecure and  = 0 otherwise].

We measured individual dietary diversity using the Individual Dietary Diversity Score (IDDS), a proxy measure of the nutritional quality of an individual's diet [24]. IDDS was calculated as the sum of the number out of the following 12 food groups consumed in the 24 hour period preceding the interview: 1) cereals; 2) roots and tubers; 3) pulses and legumes/nuts; 4) vegetables; 5) fruits; 6) meat and poultry; 7) eggs: 8) fish and seafood; 9) milk and milk products; 10) oils and fats; 11) sugar and sweets; 12) miscellaneous. We categorized IDDS into low individual dietary diversity ( = 1 if consumed <5 groups; 0 otherwise), medium dietary diversity ( = 1 if consumed 5 to 8 groups per day; 0 otherwise), and high dietary diversity ( = 1 if 9 to 12 groups per day; 0 otherwise).

In multivariate analyses, we controlled for individual, household, and community level factors that may potentially confound the relationship between HRQoL and food insecurity. Individual level variables included age (continuous), sex (male v. female), education (primary or below v. other), marital status (married/co-habiting for at least two years v. other), relationship to household head (head or spouse v. other) and CD4 count (continuous). Household level variables included household size, per capita monthly household expenditure (measured in Ugandan shillings, categorized into tertiles), and whether the homestead was within an internally displaced camp (IDP; yes/no). Community level variables included travel time to a TASO clinic in minutes, distance to the nearest government hospital in kilometers, and distance to the nearest market in kilometers, as distance to market affects food prices, access to information and resources including food. Finally, we included month and year of the interview, and a district dummy to control for district-level unobservable characteristics.

### Statistical Analysis

All data were analyzed using Stata Version 11.2 (College Station, TX). We performed descriptive analysis and multivariate linear regressions to investigate whether HFIAS and IDDS are associated with HRQoL. We first ran regressions on the two summary HRQoL outcomes, PHS and MHS. Next we ran regressions where the subscale scores were the outcomes.

For each outcome (both the summary scores and subscales), we present three sets of models. First, we present multivariate models with a dichotomous indicator for severe food insecurity ( = 1 if severely food insecure and  = 0 otherwise) as the key explanatory variable. In the second model, the primary explanatory variable is the categorical IDDS variable: low individual dietary diversity ( = 1 if consumed <5 groups; 0 otherwise); medium dietary diversity ( = 1 if consumed 5 to 8 groups per day; 0 otherwise), and high dietary diversity ( = 1 if 9 to 12 groups per day; 0 otherwise). We ran these two models separately to investigate independent associations of HFIAS and IDDS with HRQoL. The third set of models includes both HFIAS and IDDS categorical variables to assess whether the association between HFIAS and HRQoL attenuates when controlling for IDDS. We tested for multicollinearity of independent variables in all models and found no evidence of multicollinearity; therefore we included all proposed controls. Individual, household and community level characteristics elaborated above were controlled for and robust standard errors were calculated in all models.

## Results

### Sample Characteristics

Interviews were conducted with 902 individuals, and the sample size for analysis was 899. The mean Household Food Insecurity Access Scale (HFIAS) score was 3.6 (SD = 0.6) and individual dietary diversity score (IDDS) was 6.4 (SD = 1.7; Table 2). Sixty-six percent of respondents lived in households classified as severely food insecure, and the percentage of individuals experiencing low (<5 food groups/day), medium (5–8 food groups/day), and high dietary diversity (9–12 food groups per day) was 15, 76, and 9, respectively.

**Table 2:** Summary Statistics (n = 899).

Participant characteristics	Mean/percentage	Standard deviation
HFIAS Score	15.20	4.93
Individual Dietary Diversity Score	6.25	1.67
HFIAS Severity (1 = severe food insecurity)	66.07	-
Individual Dietary Diversity Low (<5 groups/day)	14.68	-
Individual Dietary Diversity Medium (5–8 good groups/day)	76.08	-
Individual Dietary Diversity High (9–12 good groups/day)	9.23	-
Age	39.09	9.61
Female	71.75	-
CD4 count (cells/mm^3^)	337.92	63.80
Education below primary level	79.42	-
Head of household or spouse	88.54	-
Married/Cohabiting ≥2 years	48.28	-
IDP camp	9.23	-
Household size	6.34	2.77
Per capita monthly expenditures	49,133	52,542
Poorest expenditure category	33.37	-
Middle expenditure category	33.37	-
Richest expenditure category	33.26	-
Time to TASO clinic (minutes)	85.75	79.86
Distance to government hospital (km)	4.05	33.32
Distance to market (km)	1.63	1.83
Interview year = 2009	67.74	-
Gulu District	49.72	-
Soroti District	50.28	-
		
Outcomes		
Physical health summary score	46.18	8.00
Mental health summary score	46.19	7.32

Cronbach's alpha for the ten subscales used to compute the PHS and MHS was 0.86, indicating a high degree of correlation between the dimensions. The mean PHS score was 46.18 (SD = 8.00), and the mean MHS score was 46.86 (SD = 7.24). Subscale scores ranged from an average of 32.29 (general health, SD = 24.93) to 82.67 (social functioning, SD = 22.49).

The average age of study participants was 39 years, and 72 percent of the sample was female (Table 2). Average CD4 count ias 338 cells/mm^3^. Education levels were low, with more than a third of the sample not having completed primary level education. Most of the study participants (89%) were either the household head or the spouse of the household head. Nine percent of the sample resided in an IDP camp, and average household size was six persons. Average per capita monthly expenditures was 49,133 Ugandan Shillings (25 USD based on exchange rate as of February 2009). Average distance to the nearest government hospital was four kilometers and to the market was 1.6 kilometers. Average travel time to a TASO clinic was 86 minutes.

### Multivariate Results, Mental Health and Physical Health Summary Scores

Results from our multivariate analysis indicated that the HFIAS and IDDS measures were correlated with MHS and PHS scores in the hypothesized directions (Table 3). PLHIVs in households with severe food insecurity had, on average, MHS scores that were 1.7 to 1.8 points lower (*p*<.001; Table 3, Columns 1 and 3) and PHS scores that were 1.5 to 1.6 points lower (*p*<.01; Table 3, Columns 4 and 6) than PLHIV in households that are not severely food insecure.

**Table 3:** Multivariate regression models of association between HFIAS, IDDS and Mental & Physical Health Summary Score (n = 899).

	(1)	(2)	(3)	(4)	(5)	(6)
	Mental health summary score	Mental health summary score	Mental health summary score	Physical health summary score	Physical health summary score	Physical health summary score
HFIAS Severity (1 = severe food insecurity)	−1.84***		−1.69***	−1.60^**^		−1.50^**^
	(−3.68)		(−3.40)	(−2.86)		(−2.70)
Individual Dietary Diversity Medium (5–8 good groups/day)		2.33^**^	2.18^**^		1.06	0.93
		(3.28)	(3.07)		(1.50)	(1.32)
Individual Dietary Diversity High (9–12 good groups/day)		3.88***	3.61***		3.04^**^	2.80^*^
		(3.96)	(3.68)		(2.70)	(2.51)
Age	−0.07^**^	−0.07^**^	−0.07^**^	−0.18***	−0.18***	−0.18***
	(−2.63)	(−2.66)	(−2.63)	(−6.49)	(−6.49)	(−6.45)
Female	−2.74***	−2.72***	−2.70***	−1.42^*^	−1.42^*^	−1.40^*^
	(−4.74)	(−4.76)	(−4.73)	(−2.31)	(−2.32)	(−2.29)
CD4 count (cells/mm^3^)	0.01^**^	0.01^**^	0.01^**^	0.01^**^	0.01^**^	0.01^**^
	(2.83)	(2.79)	(2.84)	(3.20)	(3.11)	(3.15)
Education below primary level	−0.94	−1.00	−0.79	−1.10	−1.14	−0.95
	(−1.49)	(−1.60)	(−1.25)	(−1.64)	(−1.70)	(−1.42)
Head of household or spouse	0.45	0.53	0.59	1.23	1.27	1.32
	(0.53)	(0.61)	(0.68)	(1.26)	(1.29)	(1.35)
Married/Cohabiting ≥2 years	1.18^*^	1.15^*^	1.11^*^	1.00	0.97	0.93
	(2.19)	(2.16)	(2.09)	(1.74)	(1.69)	(1.63)
IDP camp	−0.38	−0.57	−0.29	−1.73	−1.96^*^	−1.71
	(−0.44)	(−0.68)	(−0.33)	(−1.79)	(−2.04)	(−1.77)
Household size	−0.17	−0.19	−0.19	−0.08	−0.10	−0.10
	(−1.69)	(−1.88)	(−1.92)	(−0.78)	(−0.94)	(−0.96)
Middle expenditure category	−0.07	−0.15	−0.31	−0.56	−0.54	−0.68
	(−0.12)	(−0.25)	(−0.50)	(−0.85)	(−0.80)	(−1.01)
Richest expenditure category	0.91	0.77	0.57	1.19	1.12	0.94
	(1.35)	(1.15)	(0.84)	(1.62)	(1.50)	(1.25)
Time to TASO clinic (minutes)	−0.01^*^	−0.01	−0.01	−0.01^*^	−0.01^*^	−0.01^*^
	(−2.15)	(−1.85)	(−1.81)	(−2.33)	(−2.21)	(−2.18)
Distance to government hospital (km)	−0.00	0.00	0.00	0.01***	0.01***	0.01***
	(−0.52)	(0.54)	(0.66)	(3.44)	(3.72)	(3.71)
Distance to market (km)	−0.09	−0.03	−−0.04	−0.07	−0.03	−0.04
	(−0.66)	(−0.21)	(−0.31)	(−0.48)	(−0.21)	(−0.30)
Gulu District	−1.05	−0.72	−0.75	−0.60	−0.39	−0.41
	(−1.85)	(−1.27)	(−1.34)	(−0.99)	(−0.63)	(−0.68)
Constant	49.76***	46.26***	47.34***	48.73***	46.60***	47.56***
	(23.16)	(20.77)	(21.36)	(18.17)	(17.08)	(17.61)
						
R-squared	0.16	0.16	0.17	0.16	0.16	0.16

Robust t-statistics in parenthesis.

*** p<0.001, ^**^p<0.01, ^*^p<0.05.

Note: Models also control for year and month of interview.

Individual dietary diversity was positively correlated with both MHS and PHS. Individuals with high dietary diversity had MHS scores that were 3.6 to 3.9 points higher than individuals with low dietary diversity (*p*<.001; Table 3, Columns 2 and 3) and PHS scores that were 2.8 to 3.0 points higher (*p*<.05; Table 3, Columns 5 and 6). In the third set of models we included both HFIAS and IDDS categorical variables to assess whether the relationship between food security and HRQoL attenuates when including a measure of dietary diversity. We found that HFIAS remained significant in both PHS and MHS models and the regression coefficients were only mildly attenuated and still statistically significant at the one percent level (Table 3); Columns 3 and 6).

Increasing age and female sex were both negatively correlated with MHS and PHS, and CD4 count was positively correlated with both outcomes (Table 3). Education and household expenditures were not correlated with HRQoL. Being in a stable relationship was positively correlated with MHS, but not with PHS.

Severe food insecurity was significantly correlated with 9 of the 11 subscales (general health, physical functioning, role functioning, social functioning, pain, mental health, health distress, quality of life, and health transition; Table 4). High dietary diversity was significantly correlated with 7 of the 11 subscales (general health, pain, mental health, vitality, health distress, quality of life, and health transition). We found that when that dietary diversity was added to the sub-scale models, the regression coefficients for food insecurity were only minimally attenuated, and in most cases was still significant.

**Table 4:** Multivariate regression models of association between HFIAS, IDDS and MOS component scores (n = 899).

	(1)	(2)	(3)	(4)	(5)	(6)	(7)	(8)	(9)	(10)	(11)	(12)
	General health	General health	General health	Physical function-ing	Physical function-ing	Physical function-ing	Role function-ing	Role function-ing	Role function-ing	Social function-ing	Social function-ing	Social function-ing
HFIAS Severity (1 = severe food insecurity)	−4.48^*^		−4.23^*^	−2.90^*^		−2.79^*^	−3.72^*^		−3.49^*^	−4.03^**^		−3.89^*^
	(−2.48)		(−2.35)	(−2.57)		(−2.47)	(−2.28)		(−2.16)	(−2.63)		(−2.54)
Individual Dietary Diversity Medium (5–8 good groups/day)		3.65	3.28		1.15	0.90		1.68	1.38		1.40	1.05
		(1.72)	(1.55)		(0.72)	(0.56)		(0.80)	(0.66)		(0.62)	(0.47)
Individual Dietary Diversity High (9–12 good groups/day)		7.26^*^	6.57		3.47	3.02		7.29^*^	6.73^*^		4.69	4.06
		(2.00)	(1.82)		(1.38)	(1.21)		(2.20)	(2.06)		(1.48)	(1.29)
R-squared	0.14	0.13	0.14	0.20	0.20	0.20	0.09	0.09	0.09	0.11	0.10	0.11

	(13)	(14)	(15)	(16)	(17)	(18)	(19)	(20)	(21)	(22)	(23)	(24)
	Cognitive function-ing	Cognitive function-ing	Cognitive function-ing	Pain	Pain	Pain	Mental health	Mental health	Mental health	Vital-ity	Vital-ity	Vital-ity
HFIAS Severity (1 = severe food insecurity)	−1.70		−1.57	−3.19^*^		−2.86^*^	−3.43^**^		−3.19^**^	−2.12		−1.77
	(−1.31)		(−1.21)	(−2.31)		(−2.08)	(−3.06)		(−2.84)	(−1.88)		(−1.57)
Individual Dietary Diversity Medium (5–8 good groups/day)		2.30	2.16		4.72^*^	4.47^*^		4.83^**^	4.55^**^		4.81^**^	4.66^**^
		(1.30)	(1.20)		(2.54)	(2.40)		(2.97)	(2.81)		(3.29)	(3.16)
Individual Dietary Diversity High (9–12 good groups/day)		3.30	3.05		8.69^**^	8.23^**^		5.61^*^	5.09^*^		9.43***	9.15***
		(1.25)	(1.14)		(3.12)	(2.95)		(2.35)	(2.13)		(4.48)	(4.31)
R-squared	0.16	0.16	0.16	0.12	0.12	0.13	0.14	0.14	0.14	0.21	0.23	0.23

	(25)	(26)	(27)	(28)	(29)	(30)	(31)	(32)	(33)
	Health distress	Health distress	Health distress	Quality of life	Quality of life	Quality of life	Health transition	Health transition	Health transition
HFIAS Severity (1 = severe food insecurity)	−2.18^*^		−2.03	−5.02***		−4.59***	−4.22***		−3.90^**^
	(−2.07)		(−1.92)	(−3.90)		(−3.60)	(−3.46)		(−3.18)
Individual Dietary Diversity Medium (5–8 good groups/day)		3.43^*^	3.26^*^		4.60^**^	4.19^*^		4.66^**^	4.32^*^
		(2.12)	(2.01)		(2.78)	(2.56)		(2.60)	(2.42)
Individual Dietary Diversity High (9–12 good groups/day)		3.58	3.25		12.60***	11.85***		8.54^**^	7.91^**^
		(1.64)	(1.49)		(5.04)	(4.77)		(3.13)	(2.90)
R-squared	0.13	0.13	0.13	0.11	0.12	0.13	0.08	0.08	0.09

Robust t-statistics in parenthesis.

*** p<0.001, ^**^p<0.01, ^*^p<0.05.

Note: Models also controlled for age, gender, CD4 count, education, household head/spouse status, married/cohabiting, IDP camp, household size, household expenditures, time to TASO clinic (minutes), distance to government hospital (km), distance to market (km), district, interview year, and interview month.

Other covariates correlated with the subscales included age, sex (females have lower scores, on average), CD4 count (positively correlated with six subscales), distance to a government hospital (small, mixed effects), travel time to TASO clinic (negatively correlated with four subscales), and district of residence (residents from Gulu have on average higher scores on pain, quality of life and health transition and lower scores on general health, mental health, and vitality) and to a lesser extent, relationship status, residing in an IDP camp, education, expenditures, and household size (results not shown; available upon request from the authors).

## Discussion

Using a sample of 899 PLHIV in Uganda, we estimated the association between household food insecurity, individual dietary diversity, and HRQoL, controlling for disease severity and other individual, household, and community level characteristics. Our findings on the relationship between HRQoL and diet quality are novel and we highlight key results from our study below.

First, household food insecurity and poor individual dietary diversity, measured using validated scales, were correlated with lower mental and physical health summary scores; there is no evidence that the relationship between household food security and HRQoL was attenuated by including individual dietary diversity scores in the analyses. Second, compared to household food security, the association between individual dietary diversity and HRQoL measures were consistently larger. To our knowledge, our study is the first to demonstrate the association between dietary diversity and HRQoL among PLHIV. Furthermore, our study adds to the limited literature on food and nutrition security and quality of life among PLHIV in resource-poor settings. These findings suggest a possible role for interventions designed to improve food access and diet quality as part of a comprehensive package of interventions designed to mitigate the psycho-social consequences of HIV and AIDS.

Food and nutrition insecurity may affect HRQoL through different pathways, including chronic stress and nutritional status [49]. In many sub-Saharan African settings, food insecurity is the predominant form of uncertainty experienced in daily living. Given the centrality of food production and provisioning in resource limited settings, food insecurity may be strongly associated with daily and chronic stress [50]. Indeed, anxiety and uncertainty about food supply are key domains that are captured by our measure of food insecurity [48].

Heightened stress may lead to physiologic changes that influence health outcomes [51]–[53]. A growing literature documents the links between socioeconomic stress and physiological dysregulation, pathogen burden, inflammation, hormone response, and cell-mediated immunity [54]–[62]. Chronic stressors (including, but not limited to financial crisis, cut in pay, and change in job) have been found to be negatively correlated with both mental and physical health-related HRQoL among PLHIV in a developed country setting [39]. Chronic stressors may accelerate age-related declines in neuroendocrine and immune systems [63]. The body's direct response to chronic stressors, which includes stimulation of the hypothalamic-pituitary axis and the sympathetic nervous system [62], [63], can promote systemic inflammation and oxidative stress [53] which inturn could hasten disease progression [64], [65].

The second hypothesized pathway is undernutrition. The value of a diverse diet to meet the requirements for essential nutrients has long been recognized. Undernutrition weakens the immune system and may lead to increased rate of progression through compromised immunity [65]. Optimal macro- and micronutrient status of PLHIV is essential to maximize the period of asymptomatic infection, to mount an effective immune response to fight opportunistic infections, and to optimize the benefits of ART. HIV infection has long been associated with wasting syndrome, and being underweight with HIV is a strong risk factor for [66], [67], even in people receiving ART [68], [69]. The relationship between nutritional status and HRQoL is increasingly being examined among PLHIV and has even been studied in developed countries among elderly populations and those with chronic diseases [70], [71]. Many of these studies describe an association between nutritional status and varied dimensions of quality of life; however no consistent explanation of these observed associations has emerged. Weight loss and the resultant physical impairment is one mechanism of this association that has been posited [71].

In a study in the United States, Semba et al. found that improvements in hemoglobin concentrations were significantly associated with increased HRQoL among individuals living with AIDS [72], highlighting the potential of nutrition interventions to improve quality of life. Evidence from Uganda has demonstrated a strong and independent predictive link between household food security and individual dietary diversity and measures of undernutrition and survival among PLHIV [24], [34]. Extending the findings on nutritional status to HRQoL in part motivated this study. Both of the pathways through which household food security and diet quality affect HRQoL suggested above are plausible given our results. Even after controlling for other aspects of socioeconomic status (*i.e*., income and education), both food security and diet quality predicted HRQoL among PLHIV in our sample. The link between HRQoL and food insecurity may not be exclusive to PLHIV [12], [14], [15], and it is plausible that such a relationship exists among populations with other chronic diseases.

A general limitation of our study is the cross-sectional nature of our data; we cannot draw any conclusions about causality in the relationships studied. A limitation of self-reported HRQoL measures is that some aspects of HRQoL may be affected by factors in a respondent's life which are not related to treatment for HIV/AIDS or food security but nevertheless affect a respondent's mood and possibly their answers. By restricting eligibility for this study to individuals who qualified for World Food Programme rations and accessing TASO HIV care and treatment services, our findings pertaining to PLHIV may not be generalizable to a wider HIV positive population. In addition, we do not have biomarkers for nutrient deficiencies in our data. Finally, our study design cannot account for unobserved confounders, and this limitation may bias our estimate of the associations between HRQoL and food insecurity measures.

Implications for future research arise from this study. First, research should focus on understanding the different physiological, psychological, and behavioral pathways through which food security and dietary quality influence HRQoL among PLHIV and other populations with chronic diseases. Second, longitudinal data and rigorous impact evaluations are needed that examine changes in different HRQoL domains and mental and physical health summary scores, with different intervention modalities; little is known about what food and nutrition interventions specifically influence improvements in HRQoL. This focus on implementation science is best positioned to inform programming and policy decisions and to ensure that outcomes of mental health are included in HIV care and treatment program objectives, alongside more traditional HIV-related outcomes, particularly in developing country settings.

In summary, our findings demonstrate a link between food security, diet quality, and HRQoL that could influence food and nutrition security programming within the context of HIV/AIDS. Conventional food and nutrition security interventions targeted to PLHIV have the stated goals of ensuring nutritional recovery and treatment success through nutrition and/or food support; and mitigating the effects of AIDS on individuals and households through safety nets [73]. Little to no consideration is given to the potential of these interventions for improving quality of life, by all accounts, an important consequence that HIV has on everyday behavior, social activities and psychological wellbeing and disease progression. Including HRQoL as a stated goal should be considered as part of a more holistic approach to food and nutrition security programming within the context of HIV. Our results warrant careful consideration of the ability of traditional food security interventions–food, cash, or voucher transfer–to bring about resultant improvements in diet quality in isolation. Both the composition of the actual intervention and the operating environment within which these interventions operate are critical considerations that need to be incorporated into program designs if intended outcomes such as improvements in diet quality are to be achieved.
